# Resveratrol for Easing Status Epilepticus Induced Brain Injury, Inflammation, Epileptogenesis, and Cognitive and Memory Dysfunction—Are We There Yet?

**DOI:** 10.3389/fneur.2017.00603

**Published:** 2017-11-13

**Authors:** Olagide W. Castro, Dinesh Upadhya, Maheedhar Kodali, Ashok K. Shetty

**Affiliations:** ^1^Olin E. Teague Veterans’ Medical Center, Central Texas Veterans Health Care System, Temple, Texas, United States; ^2^Institute for Regenerative Medicine, Department of Molecular and Cellular Medicine, Texas A&M Health Science Center College of Medicine, College Station, Texas, United States; ^3^Institute of Biological Sciences and Health, Federal University of Alagoas (UFAL), Maceio, Brazil; ^4^Department of Anatomy, Kasturba Medical College, Manipal University, Manipal, India

**Keywords:** epilepsy, seizures, memory impairment, neuroprotection, GABA-ergic interneurons, hippocampal neurogenesis, neuroinflammation, oxidative stress

## Abstract

Status epilepticus (SE) is a medical emergency exemplified by self-sustaining, unceasing seizures or swiftly recurring seizure events with no recovery between seizures. The early phase after SE event is associated with neurodegeneration, neuroinflammation, and abnormal neurogenesis in the hippocampus though the extent of these changes depends on the severity and duration of seizures. In many instances, over a period, the initial precipitating injury caused by SE leads to temporal lobe epilepsy (TLE), typified by spontaneous recurrent seizures, cognitive, memory and mood impairments associated with chronic inflammation, reduced neurogenesis, abnormal synaptic reorganization, and multiple molecular changes in the hippocampus. While antiepileptic drugs are efficacious for terminating or greatly reducing seizures in most cases of SE, they have proved ineffective for easing SE-induced epileptogenesis and TLE. Despite considerable advances in elucidating SE-induced multiple cellular, electrophysiological, and molecular changes in the brain, efficient strategies that prevent SE-induced TLE development are yet to be discovered. This review critically confers the efficacy and promise of resveratrol, a phytoalexin found in the skin of red grapes, for easing SE-induced neurodegeneration, neuroinflammation, aberrant neurogenesis, and for restraining the evolution of SE-induced brain injury into a chronic epileptic state typified by spontaneous recurrent seizures, and learning, memory, and mood impairments.

## Introduction

Status epilepticus (SE) is a medical emergency exemplified by continuous tonic-clonic seizure activity lasting five or more minutes or a series of seizures with no recovery between them (1). The incidence of SE varies from 10 to 61 per 100,000 population each year. The frequency of SE is higher in children and the aged population, and the overall SE-related mortality is ~20% (2–4). SE can occur from multiple causes, including head injury, febrile seizures, stroke, brain infections, sleep deprivation, withdrawal from alcohol and drugs of abuse, or pre-existing conditions, such as brain tumor, congenital malformations, and Alzheimer’s disease. Although a combination of antiepileptic drugs (AEDs) terminate seizures in most cases of SE, the first line of AEDs, such as benzodiazepines and phenytoin are ineffective for ceasing seizures in 30–40% of SE cases (2, 5, 6). Moreover, AEDs have undesirable side effects and do not positively modulate the pathological sequelae of SE. Indeed, significant numbers of SE-survivors display morbidity characterized by cognitive, memory, and mood dysfunction with an enhanced risk for developing chronic temporal lobe epilepsy (TLE). Hence, alternative therapies, alone or in combination with AEDs, are necessary for reducing SE-induced mortality, as well as easing SE-induced pathological ramifications, such as neurodegeneration; neuroinflammation; abnormal hippocampal neurogenesis; epileptogenesis; cognitive, memory, and mood dysfunction; and chronic TLE.

The hippocampus is one of the highly susceptible regions of the brain to be inflicted with SE-induced injury and for developing enduring pathological alterations in structure and function (7, 8). For example, in the early phase after SE elicited by chemo-convulsants, such as the kainic acid (KA) or pilocarpine, degeneration of some dentate hilar neurons and CA1 and CA3 pyramidal neurons is consistently seen in the hippocampus (9–13). Moreover, such neurodegeneration is associated with increased as well as aberrant neurogenesis (14–19). SE enhances neural stem cell (NSC) proliferation in the hippocampus, which is likely triggered by the release of NSC mitogenic factors from dying neurons, deafferented granule cells, and reactive glia (20–22) and elevated gamma-amino butyric acid (GABA) levels (23). These alterations cause increased neurogenesis as well as an abnormal migration of newly born neurons to the dentate hilus (DH) and the dentate molecular layer (ML). The addition of greater numbers of new neurons to the granule cell layer (GCL) after SE has been recognized to be beneficial due to their reduced excitability feature (24). However, abnormal migration of a substantial number of newly born neurons has been suggested to be detrimental due to their propensity for forming epileptogenic circuitry (17, 25–28).

The hippocampal neurodegeneration resulting from SE typically ensues with persistently increased oxidative stress and inflammation (29–32), declined neurogenesis (16, 33, 34), and aberrant sprouting of dentate granule cell axons (mossy fibers) into the inner ML of the dentate gyrus (DG) (35–39). Furthermore, learning and memory impairments (40–44), loss of calbindin expression in dentate granule cells and CA1 pyramidal neurons (45, 46), alterations in neurotransmitter and other receptors (47–49), and functional modifications in astrocytes (50) also occur. Considerably waned neurogenesis in the chronic phase after SE appears to be due to an altered fate-choice decision of newly born cells with a preference to differentiate into glia rather than neurons, likely due to changes in the neurotrophic milieu of neurogenic niches (16, 22, 51) and continued inflammation (30, 31, 44). Importantly, a greater portion of residual neurogenesis in the chronic phase remains aberrant with much of newly born neurons migrating into the DH or giving rise to basal dendrites (34). Both decreased and abnormal neurogenesis in the chronic phase likely contribute to learning, memory, and mood impairments, in addition to enhancing epileptogenic circuitry. Aberrant mossy fiber sprouting is another prominent structural change in the hippocampus after SE. Many studies have suggested that aberrant mossy fiber sprouting is one of the major causes of TLE (52–54). Although there is no consensus on this issue, there is enough evidence to believe that aberrant mossy fiber sprouting contributes at least some extent to the frequency and/or intensity of spontaneous recurrent seizures (SRS) in TLE (55–58).

Thus, multiple epileptogenic and neurogenic changes contribute to the progression of SE-induced injury into chronic epilepsy, typified by SRS, and learning, memory, and mood impairments (59–62). However, the manifestation of TLE in humans following SE may take months, years, or even decades, depending on the degree and the swiftness by which the various epileptogenic changes achieve required ceilings to produce hippocampal hyperexcitability. The delay provides a large window to intervene with promising alternative drugs or natural compounds that are efficacious for alleviating oxidative stress, inflammation, and abnormal neurogenesis. While intervention in the early phase after SE may considerably reduce neurodegeneration, intervention in both early and latent phases or in the latent phase alone may block or slow-down the subsequent epileptogenic changes, thwart or delay the development of SRS and/or prevent cognitive, memory, and mood impairments. However, it is important to note that partial neuroprotection or limited suppression of ensuing inflammation provided by drugs does not necessarily prevent epileptogenesis and/or the related comorbidities. For example, anticonvulsant drugs, such as dizocilpine and retigabine, interleukin-1 receptor antagonist anakinra, interleukin-1β inhibitor VX765, or a melatonin receptor agonist agomelatine, can provide partial protection against SE-induced neuron loss and inflammation but cannot prevent epileptogenesis (63–65). This discrepancy reflects the fact that neurodegeneration and neuroinflammation are not the exclusive reasons driving epileptogenesis and the occurrence of SRS after SE or brain injury (66). Therefore, it is imperative to identify drugs that not only provide neuroprotection and suppress inflammation but also reduce multiple other epileptogenic changes after SE. In addition, it will be essential to determine for every candidate drug, whether short-term treatment in the immediate post-SE period or prolonged treatment for several weeks after SE is needed. In this review, we focus on discussing the promise of resveratrol (RESV), a phytoalexin found in the skin of red grapes, certain berries, and peanuts, for easing SE-induced neurodegeneration, neuroinflammation, epileptogenesis, chronic seizures and the associated comorbidities.

## Source of RESV and Its Known Biological Activities

Resveratrol (3,5,4′-trihydroxystilbene) is a natural phytoalexin, produced by grapevines, pines, and legumes in response to bacterial or fungal infections, injury, or stress (67, 68). RESV is also found in raspberries, mulberries, plums, peanuts, bilberries, blueberries, cranberries, Scots pine, and Japanese knotweed. A vast majority of these sources contain both cis- and trans-isomeric forms of RESV. However, the trans-isomer has received the utmost attention because of its role in beneficial effects of RESV (69).

Numerous studies have shown that RESV facilitates a range of biological activities, including longevity and prevention of cancer (70–72). Studies in animal models of human diseases have pointed out that RESV has anti-ischemic, antiviral, antioxidant, and antiinflammatory properties (73–76). Besides, RESV has been shown to delay several age-related diseases (70, 77, 78). Its neuroprotective property has been seen in several cell culture models (79, 80) as well as *in vivo* models of neuroinflammation (81), stroke (82), spinal cord injury (83), multiple sclerosis (84), Huntington’s disease (85), and traumatic brain injury (86). The other features of RESV that are attractive for therapeutic use include its ability to cross the blood–brain barrier after peripheral administration, its minimal side effects and its prolonged activity in the brain (~4 h) after peripheral administration (87–89).

## Results from Clinical Trials Regarding the Beneficial Effects of RESV on Human Health

The effects of RESV on insulin sensitivity has been somewhat controversial. Two clinical trials in obese humans and type 2 diabetes patients demonstrated that 4-weeks of RESV treatment improved insulin sensitivity associated with reductions in low-level inflammation, blood pressure, and liver fat accumulation (80–91). However, other clinical trials showed no such improvement in non-obese women with normal glucose tolerance (92) and obese healthy men (93). Discrepancies in results between these studies have been attributed to differences in study designs, populations, and resveratrol formulations (94). It is also likely that RESV is not efficacious for enhancing glucose handling in subjects where normal glucose homeostasis is already maintained but effective in subjects suffering from insulin resistance. In line with this notion, a recent clinical trial showed that 4 months of RESV treatment in middle-aged men with metabolic syndrome could induce increased muscle turnover, lipid metabolism, and accumulation of long-chain saturated, monounsaturated, and polyunsaturated free fatty acids, and beneficial alterations in gut microbiota (94). Another recent clinical trial showed that incorporation of RESV to standard antihypertensive treatment is adequate for reducing blood pressure to normal levels, without the need for additional antihypertensive drugs (95). This study also implied prevention of liver damage with RESV intake, based on lower levels of hepatic enzyme glutamate-pyruvate transaminase in the serum.

Several clinical trials have also suggested that RESV treatment is beneficial for improving human brain function. For instance, improved memory performance allied with enhanced hippocampal functional connectivity between the hippocampus and the medial prefrontal cortex has been observed with RESV treatment in healthy overweight elderly individuals (96). RESV has also been shown to enhance neurovascular coupling and cognitive performance in type 2 diabetes patients (97). Furthermore, in individuals with mild to moderate Alzheimer disease, RESV treatment modulated amyloid β-40 levels in both plasma and cerebrospinal fluid, in comparison to the placebo-treated group (98). Overall, clinical studies conducted so far imply that RESV is safe, well-tolerated and beneficial with minimal side effects. Nonetheless, detailed, long-term follow-up studies are needed to fully understand the efficacy of RESV for improving the health in people with brain diseases.

## Potential Mechanisms Underlying the Neuroprotective Properties of RESV

Many studies have suggested that RESV mediates protective effects through its robust antioxidant and antiinflammatory activities, more so in aging or disease conditions (32, 99–101). First, RESV can reduce reactive oxygen species (ROS) generation through several mechanisms. It can suppress mitochondrial complex III activity, and the release of cytochrome *C* (102, 103) as well as modulate *N*-methyl-d-aspartate, α-amino-3-hydroxy-5-methyl-4-isoxazolepropionic acid or KA receptor, and intracellular Ca^2+^ pathway. RESV can also prevent mitochondrial dysfunction and impairments in Na^+^K^+^-ATPase activity after glutamate activation (104). Second, RESV can inhibit lipid peroxidation and enhance antioxidant and heme oxygenase 1 activity (89, 105). Third, RESV can restrain the loss of proteins that are implicated in cognitive disorders (106), stimulate AMP kinase activity and mitochondrial biogenesis (107), and dampen the increased electrical activity of neurons (108). Fourth, RESV can indirectly mediate beneficial effects through activation of sirtuin 1 (SIRT1). SIRT1, a class III histone deacetylase, can regulate multiple biological activities, including oxidative stress, inflammation, cellular senescence, autophagy, apoptosis, differentiation, stem cell pluripotency, metabolism, and mitochondrial biogenesis (109). In the brain, SIRT1 can influence chromatin remodeling, DNA repair, cell survival, neurogenesis, synaptic plasticity, and memory (110, 111). Finally, RESV can mediate antiinflammatory actions through activation of AMP kinase and subsequent inhibition of mammalian target of rapamycin pathway. These, in turn, inhibit the activation of nuclear factor-kappa B and the production of proinflammatory molecules induced by SE (112).

## Effects of RESV Pre-Treatment on Excitotoxic Brain Injury or Acute Seizures

Neuroprotective effects of RESV has been seen in several animal models of excitotoxic brain injury. Most of the earlier studies have, however, examined the effects of pre-treating animals with RESV on subsequent neurodegeneration mediated by excitotoxic agents. Numerous beneficial effects were observed with a variety of pre-treatment approaches. While one study showed reduced brain damage with KA administration after chronic RESV pre-treatment (113), another study demonstrated significant protection against KA-induced seizures and increased oxidative stress with the administration of RESV performed 5 min before KA treatment (114). Moreover, delayed onset of the epileptiform electroencephalographic (EEG) discharges, and reduced malondialdehyde (MDA, a byproduct of lipid peroxidation) levels were observed with RESV treatment occurring 30 min before intracortical placement of ferric chloride (115). Additional studies demonstrated neuroprotection against pilocarpine-induced SE with RESV administration occurring 30 min before pilocarpine treatment (112) and reduced seizure activity and mortality with 6 weeks of RESV treatment prior to KA administration (116). Furthermore, increased latency to myoclonic jerks and seizures, decreased number of myoclonic jerks, reduced neuronal injury, oxidative stress, and apoptosis were observed in Wistar rats receiving RESV 30 min prior to pentylenetetrazole (PTZ)-induced kindling (135). A subsequent study also showed that concurrently treating animals with KA and RESV (daily for 5 days) results in significant neuroprotection (117).

Multiple mechanisms have been suggested for the neuroprotective effects of RESV pre-treatment against seizures. A few studies showed that RESV moderates excitatory synaptic neurotransmission *via* inhibition of the voltage-gated potassium currents, and post-synaptic glutamate receptors (108, 118). The other studies showed the ability of RESV for suppressing reactive astrocytes and activated microglia (117), scavenging and opposing the production of ROS, antioxidant, antiapoptotic, and antiinflammatory activity (102, 112, 116, 119). Considering the effects of RESV on excitatory synaptic neurotransmission and post-synaptic glutamate receptors, it is plausible that RESV pre-treatment impacts the overall SE activity, which likely influences pathogenesis that follows SE. However, detailed EEG studies on the intensity of SE in RESV pre-treated animals vis-à-vis untreated animals are lacking. Although the beneficial effects of RESV pre-treatment or concurrent treatment of excitotoxins and RESV in different animal models are useful for understanding mechanisms by which RESV mediates neuroprotection, there is little translational value with this approach. Pre-treatment approach may, however, be relevant to a smaller percentage of people who take RESV daily as an antioxidant or antiinflammatory dietary supplement. However, it remains to be determined whether such small daily doses would be adequate to have protective effects against brain insults.

## Efficacy of RESV Treatment Commencing after the Onset of SE on Seizure-Induced Neurodegeneration, Neuroinflammation, and Abnormal Neurogenesis

**Figure 1:**
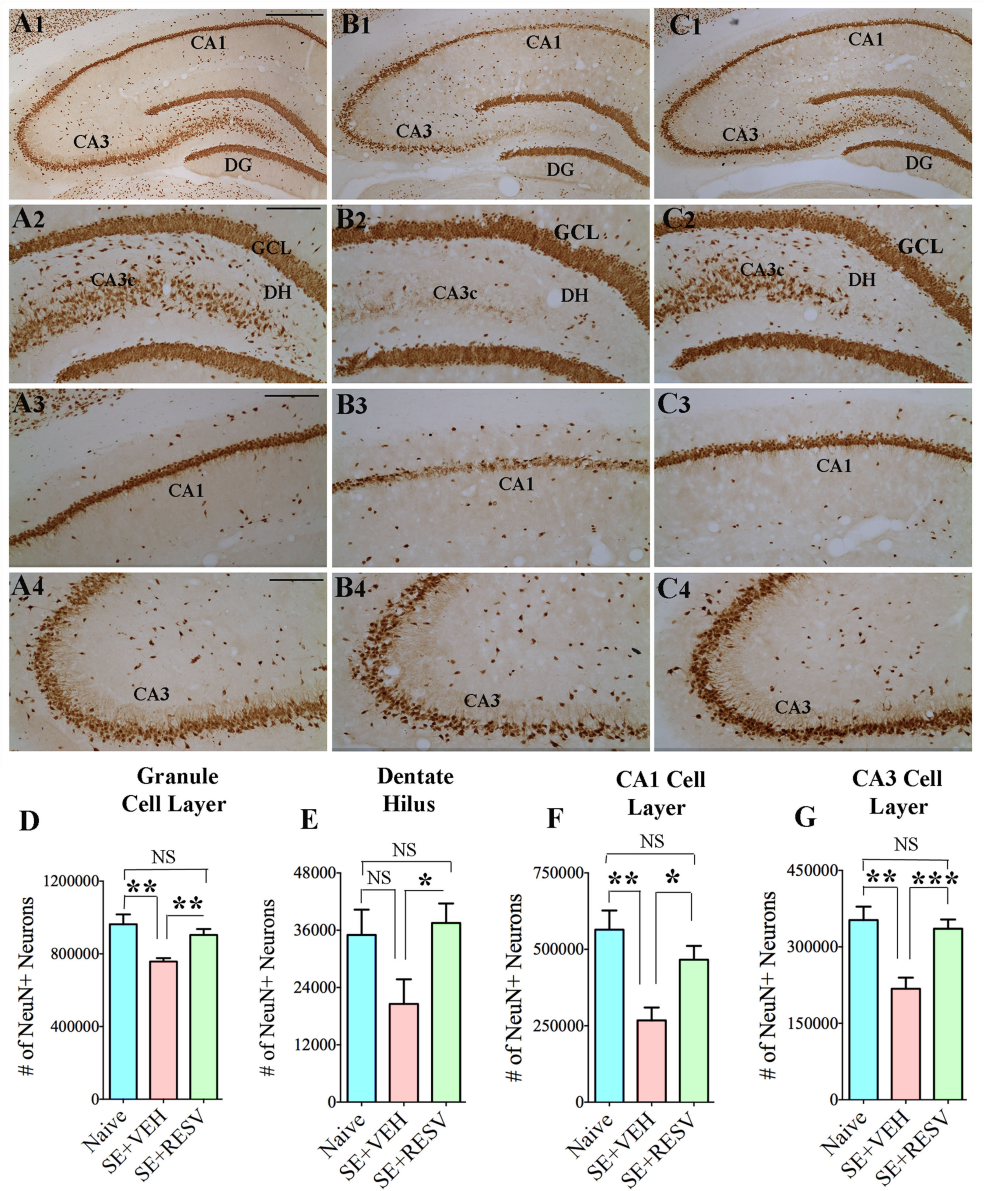
Four days of resveratrol (RESV) treatment after status epilepticus (SE) considerably curtailed neuron loss in the dentate hilus (DH), granule cell layer (GCL), and hippocampal CA1 and CA3 pyramidal cell layers. Panels **(A1–C4)** illustrate neuron-specific nuclear antigen (NeuN) positive neurons in different subfields of the hippocampus from a naïve control animal **(A1–A4)**, an animal receiving vehicle (VEH) after SE **(B1–B4)**, and an animal receiving RESV after SE **(C1–C4)**. Scale bar, **(A1,B1,C1)**, 500 µm; **(A2–A4,B2–B4,C2–C4)**, 200 µm. Bar charts display numbers of NeuN + neurons in distinct hippocampal cell layers. Animals receiving VEH after SE displayed considerably diminished numbers of neurons in the DH **(D)**, GCL **(E)**, and the CA1 and CA3 pyramidal cell layers **(F,G)**. However, neuron numbers were comparable between animals receiving RESV after SE and naïve control animals **(D–G)**, implying robust neuroprotection by RESV. *p < 0.05; **p < 0.01; ***p < 0.001; NS, not significant. Figure reproduced from Mishra et al. (32).

**Figure 2:**
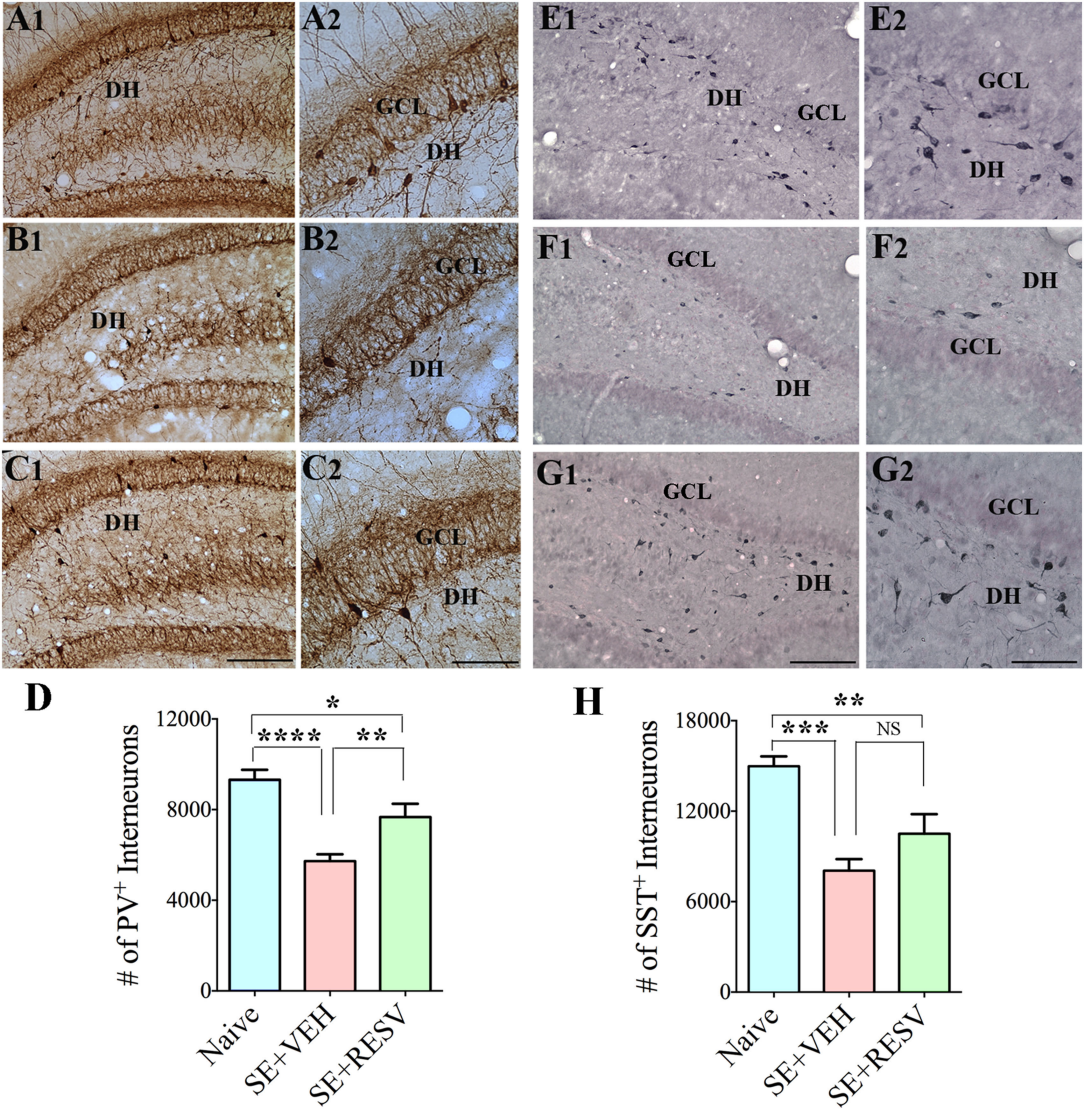
Four days of resveratrol (RESV) treatment after status epilepticus (SE) restrained the loss of inhibitory interneurons expressing the calcium-binding protein parvalbumin (PV) and the neuropeptide somatostatin (SST) in the dentate gyrus (DG). **(A1–C2)** and **(E1–G2)**, respectively, illustrate PV+ interneurons and SST+ interneurons in the DG from a naïve control animal **(A1–A2**, **E1–E2)**, an animal receiving vehicle (VEH) after SE **(B1–B2,F1–F2)**, and an animal receiving RESV after SE **(C1–C2,G1–G2)**. DH, Dentate hilus; GCL, granule cell layer. Scale bar: **(A1,B1,C1,E1,F1,G1)**, 200 µm; **(A2,B2,C2,E2,F2,G2)**, 100 µm. Bar charts display numbers of PV+ **(D)** and SST+ **(H)** interneurons in the DG. Animals receiving VEH after SE displayed considerable loss of PV+ and SST+ interneurons. However, animals receiving RESV showed only modest loss of PV+ and SST+ interneuron numbers. *p < 0.05; **p < 0.01; ***p < 0.001; ****p < 0.0001. Figure reproduced from Mishra et al. (32).

**Figure 3:**
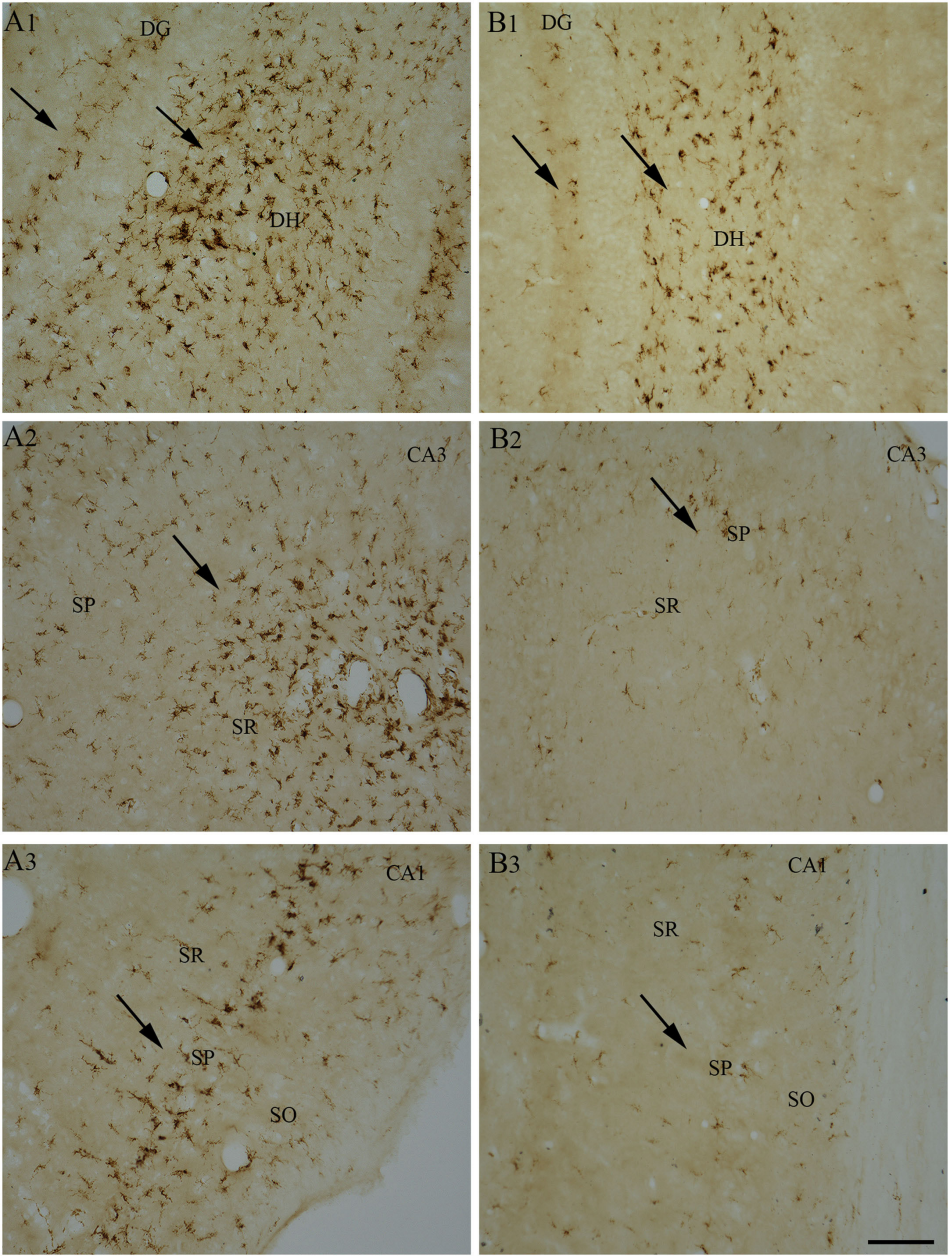
Four days of resveratrol (RESV) treatment after status epilepticus (SE) reduced the signs of inflammatory processes in the dentate gyrus (DG) and CA1 and CA3 subfields. A robust inflammation, indicated by large numbers of ED-1+activated microglia, is obvious in the DG, CA1, and CA3 hippocampal subfields of the animal receiving vehicle after SE **(A1–A3)**, in comparison to a reduced inflammation in the animal receiving RESV after SE **(B1–B3)**. SO, Stratum oriens. SP, Stratus pyramidale, SR, Stratus radiatum. Scale bar: **(A1–B3)**, 100 µm.

So far, only a few studies have analyzed the effects of RESV treatment starting hours after the onset of SE on SE-induced detrimental effects. A recent study, using a graded intraperitoneal KA administration model of SE provided the first proof that RESV treatment starting 1 h after SE onset was effective for considerably restraining SE-induced hippocampal damage (32). In this study, 4 days of RESV treatment (3 hourly doses on SE day commencing 1 h after SE, twice daily doses on SE days 2–4) was found efficacious for providing robust neuroprotection against SE. In comparison to animals receiving vehicle after SE, animals receiving RESV after SE demonstrated robust preservation of glutamatergic neurons in the GCL, DH, and CA1 and CA3 subfields of the hippocampus (Figure 1), and greater levels of maintenance of subclasses of GABA-ergic interneurons expressing parvalbumin, somatostatin (Figure 2), and neuropeptide Y. Moreover, RESV treatment after SE resulted in normalization of seizure-induced increased oxidative stress. This was evidenced in RESV treated rats by the maintenance of hippocampal MDA and the expression of multiple genes related to oxidative stress response to levels seen in naïve control animals. Controlling oxidative stress after SE has great significance as greatly elevated oxidative stress can facilitate progressive neuron loss and impair the function of remaining neurons. Indeed, increased levels of MDA has been seen in epileptic patients (120, 121). However, in a seizure study using neonatal animals, RESV therapy did not restrain SE-induced brain damage because SE in neonatal animals does not elevate oxidative stress (122). From this perspective, it is noteworthy that RESV therapy for SE is effective only in conditions where elevated oxidative stress is one of the major initial sequels of SE. Thus, RESV therapy for SE is likely more suitable for the adult and aged populations where oxidative stress is among the prominent initial pathological changes. Furthermore, 4 days of RESV therapy was effective for suppressing SE-induced inflammation in the hippocampus. This was evinced mainly through reduced concentration of tumor necrosis factor-alpha protein and diminished numbers of activated microglia in the hippocampus (Figure 3) but did not involve modulation of nuclear factor-kappa B or SIRT1 activity (32).

**Figure 4:**
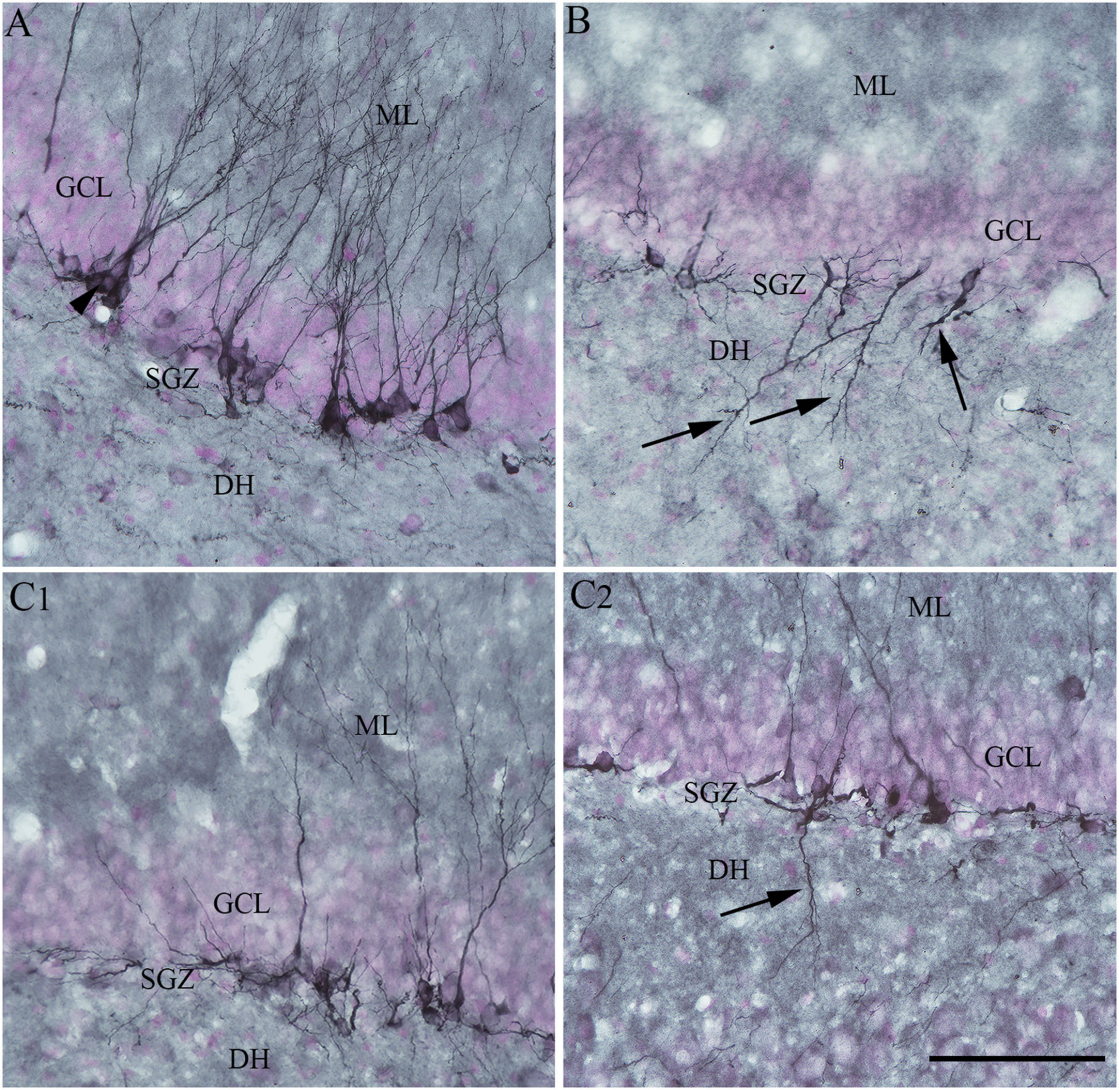
Four days of resveratrol (RESV) treatment after status epilepticus (SE) restrained the dendrites of newly born neurons projecting into the dentate hilus (DH). In the naïve control animal **(A)**, the normal polarity of newly born neurons is apparent from virtually all dendrites projecting into the molecular layer (ML) of the dentate gyrus. Contrastingly, in the animal receiving vehicle after SE **(B)**, a significant number of newly born neurons showed either abnormal polarity with apical dendrites projecting into the dentate hilus (DH; arrows) or basal dendrites projecting into the hilus. Interestingly, in animals receiving RESV after SE **(C1,C2)**, there were no newly born neurons with apical dendrites projecting into the dentate hilus **(C1)**. In addition, the occurrence of newly born neurons with basal dendrites projecting into the hilus was reduced [arrow in **(C2)**]. Scale bar = 100 µm.

Moreover, RESV treatment after SE in the above study reduced aberrant neurogenesis (Figure 4). Both numbers of newly born neurons that migrated abnormally into the DH and occurrences of basal dendrites from newly born neurons were diminished. Reduced aberrant neurogenesis mediated by RESV has functional implications because such neurogenesis promotes epileptogenic circuitry between ectopically placed granule cells and the CA3 pyramidal neurons, and between basal dendrites of granule cells projecting into the DH and granule cell axons (mossy fibers). These abnormal synaptic connectivities may contribute to occurrences of SRS in the chronic phase after SE (25–28, 123–126). In summary, the above study provided novel proof that RESV treatment starting 1 h after SE is also favorable for curtailing SE-caused elevated oxidative stress, neuron loss, inflammatory cascade, and anomalous neurogenesis, all of which can contribute to the development of a chronic epileptic state after SE.

## Efficacy of RESV Treatment for Easing Epileptogenesis and Chronic Epilepsy

**Figure 5:**
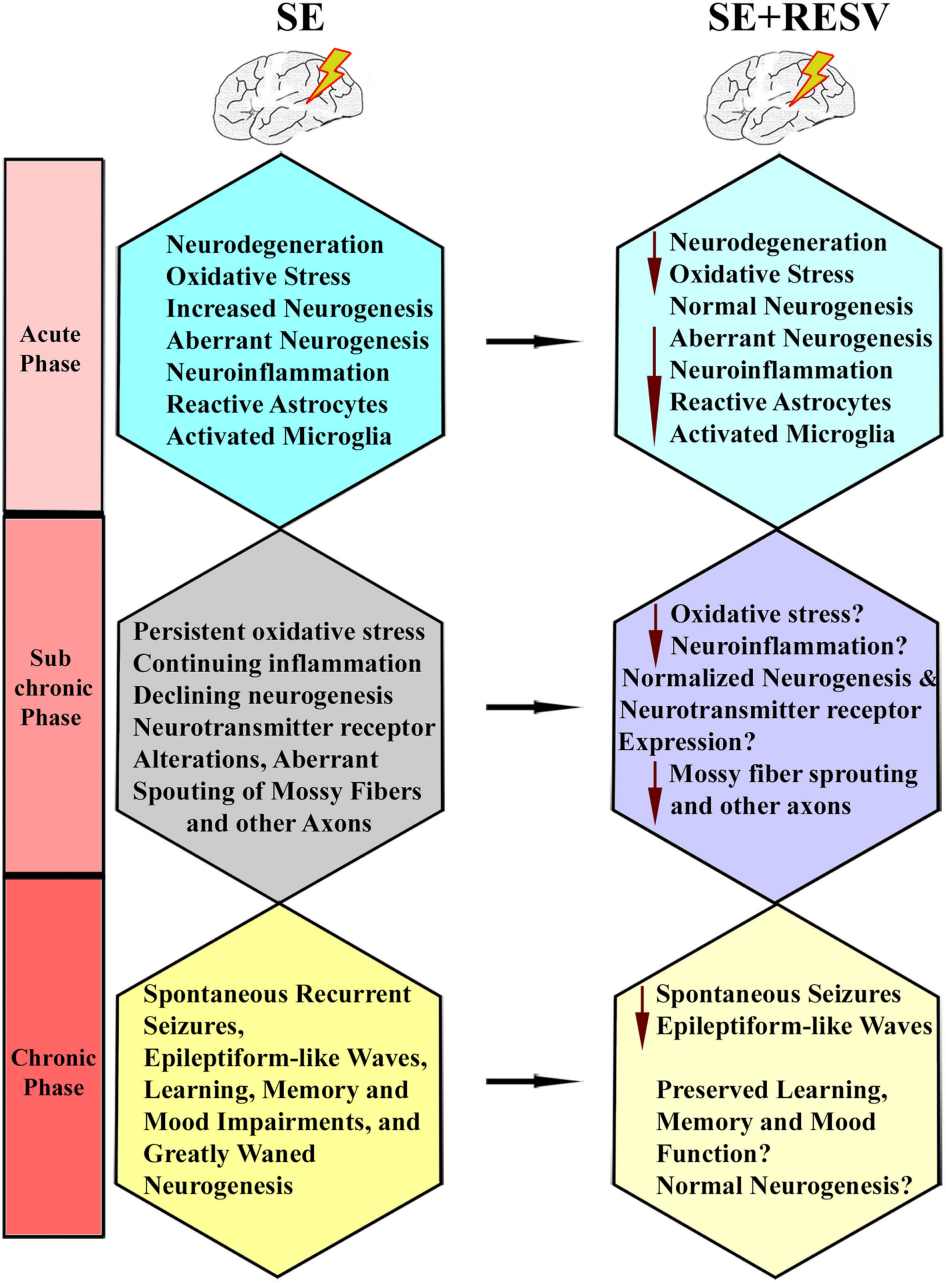
Illustration of how resveratrol (RESV) treatment after status epilepticus likely restrains or modifies the various detrimental effects of SE in the acute, subchronic, and chronic phases.

A study by Wu and colleagues examined the effects of oral administration of RESV for 10 days after an intrahippocampal injection of KA into anesthetized rats (127). They found that a reduced percentage of animals receiving RESV after KA displayed behavioral SRS at 9-weeks post-KA, in comparison to rats receiving KA alone. Besides, 2 h of EEG recordings showed diminished epileptiform-like waves in rats receiving RESV after KA, associated with some neuroprotection in the CA1 and CA3a cell layer and reduced aberrant mossy fiber sprouting into the dentate supragranular layer. These results suggest that RESV treatment starting after the induction of hippocampal injury has the potential for reducing the incidence and intensity of injury-induced chronic epilepsy. However, there are several caveats in this study. Since KA was administered directly into the hippocampus under chloral hydrate anesthesia, the influence of anesthesia on the intensity of SE is an issue. Furthermore, direct application of KA caused considerable neurodegeneration in the DH and CA3b and CA3c subregions, likely due to localized excitotoxicity. Hence, the neuroprotective effect of RESV that commenced after KA injection could not be ascertained on dentate hilar and CA3b and CA3c pyramidal neurons. Also, SRS and epileptogenic changes were measured only in the early phase after KA administration during which minimal SRS are seen. Another study evaluated the effects of RESV on a few behavioral and pathological changes in a rat model of epilepsy induced *via* PTZ kindling (128). The results suggested improved cognitive function, diminished neuronal loss in CA1 and CA3 hippocampal subfields, and reduced S100-beta protein levels in the cerebrospinal fluid and serum, in animals receiving RESV after PTZ. Furthermore, a recent study showed that acute RESV treatment after an intrahippocampal injection of KA partially inhibits evoked epileptiform discharges in the hippocampal CA1 region (129). Additional analyses demonstrated that long-term RESV treatment in this model could normalize the expression of hippocampal kainate glutamate receptors and the GABA_A_ receptor alpha1 subunit, and inhibit the KA-induced increased glutamate/GABA ratio in the hippocampus (129). Overall, the results of a few studies on anti-epileptogenic effects of RESV are promising but not conclusive. Additional rigorous longitudinal studies in animal models of epilepsy on specific epileptogenic changes are required to understand the extent to which RESV treatment can curtail epileptogenic changes that ensue after SE. Figure 5 illustrates the known and likely beneficial effects of early RESV treatment after SE on the various detrimental sequelae in the acute, subchronic, and chronic phases.

## Conclusion and Future Directions

From the results of pre-clinical studies performed so far, RESV appears to be a promising compound to employ as an adjunct to AED therapy for SE. The idea is to use AEDs for terminating SE and then employ RESV for prolonged periods to block or lessen SE-induced maladaptive structural changes as well as the development of epileptogenic circuitry. Nonetheless, additional studies are critically required prior to clinical application to ascertain whether the amount of neuroprotective, antioxidant, antiinflammatory, and normal-neurogenesis promoting effects offered by RESV therapy after SE is adequate for thwarting or at least greatly restraining the progression of SE-induced hippocampal injury into a chronic epileptic state exemplified by SRS and cognitive, memory, and mood impairments. Particularly, the following issues need to be addressed. First, SRS develop progressively after an incidence of SE and typically require 3–5 months of time to exhibit a consistent frequency and intensity of SRS over weeks (12, 13, 130, 131). Therefore, to determine the benefits of RESV administration after SE, detailed analyses of SRS through chronic EEG recordings are required at 3–5 months post-SE. Second, validation of the efficacy of RESV for preventing or greatly restraining SE-induced epileptogenic changes and the associated comorbidities will be required at different time-points after SE. These should comprise quantification of the (i) progressive loss of both glutamatergic neurons and GABA-ergic interneurons in the hippocampus and the various extrahippocampal regions; (ii) continued abnormal migration of newly generated neurons into the hilus and the ML of the DG, in relation to the survival of reelin-positive interneurons that aid the migration of newly born neurons (32, 44); (iii) extent of abnormal synaptic reorganization of dentate mossy fibers and entorhinal axons (37, 132); (iv) alterations in astrocyte function (133, 134); (v) progression of neuroinflammation (30, 31); (vi) attainment of chronic epileptic state typified by SRS; (vii) cognitive and memory impairments; and (viii) extent of depression. Third, it will be important to identify the time-point after SE at which commencement of RESV treatment provides maximal neuroprotection and prevents the progression of SE-induced brain damage into a state typified by SRS and cognitive, memory, and mood impairments. Fourth, it will be necessary to know the effects of different doses of RESV treatment occurring for shorter periods after SE (e.g., 4–14 days of treatment) and continuing for longer durations after SE (e.g., 3–12 weeks) on outcomes such as SRS frequency and intensity, and cognitive, memory and mood function. Furthermore, determining the best route of RESV administration after SE for achieving maximal efficacy with minimal side effects will be important. Routes of administration that are clinically practical for repeated administration may be examined, which may include oral and intranasal routes. Finally, any potential adverse effects of long-term administration of higher doses of RESV after SE need to be examined.

## Author Contributions

OC and DU wrote the first draft of the manuscript text and prepared figures. MK provided input to the first draft and prepared the reference list. AS edited and prepared the final version of the manuscript text and figures.

## Disclaimer

Department of Veterans Affairs, Department of Defense, United States Government Disclaimer: The contents of this article suggest the views of authors and do not represent the views of the U.S. Department of Veterans Affairs, Department of Defense or the United States Government.

## Conflict of Interest Statement

The authors declare that the research was conducted in the absence of any commercial or financial relationships that could be construed as a potential conflict of interest.
